# Smoking, Alcohol Intake and Torque Teno Virus in Stable Kidney Transplant Recipients

**DOI:** 10.3390/v15122387

**Published:** 2023-12-06

**Authors:** Caecilia S. E. Doorenbos, Jip Jonker, Jiasi Hao, Edmund J. Gore, Daan Kremer, Tim J. Knobbe, Anoek A. E. de Joode, Jan Stephan F. Sanders, Olivier Thaunat, Hubert G. M. Niesters, Coretta C. Van Leer-Buter, Stephan J. L. Bakker

**Affiliations:** 1 Department of Internal Medicine, Division of Nephrology, University Medical Center Groningen, University of Groningen, Hanzeplein 1, 9713 GZ Groningen, The Netherlands; j.jonker02@umcg.nl (J.J.); 2 Department of Epidemiology, University Medical Center Groningen, University of Groningen, Hanzeplein 1, 9713 GZ Groningen, The Netherlands; 3 Department of Medical Microbiology, Division of Clinical Virology, University Medical Center Groningen, University of Groningen, Hanzeplein 1, 9713 GZ Groningen, The Netherlands; 4 Department of Transplantation Nephrology and Clinical Immunology Hospices Civils de Lyon, Claude Bernard Lyon I University, INSERM Unit 1111, 69003 Lyon, France

**Keywords:** Torque Teno Virus, smoking, alcohol, immunosuppression, kidney transplantation

## Abstract

Torque Teno Virus (TTV) is a non-pathogenic virus that is highly prevalent among kidney transplant recipients (KTRs). Its circulating load is associated with an immunological status in KTR and is considered a promising tool for guiding immunosuppression. To allow for optimal guidance, it is important to identify other determinants of TTV load. We aimed to investigate the potential association of smoking and alcohol intake with TTV load. For this cross-sectional study, serum TTV load was measured using PCR in stable kidney transplant recipients at ≥1 year after transplantation, and smoking status and alcohol intake were assessed through questionnaires and measurements of urinary cotinine and ethyl glucuronide. A total of 666 KTRs were included (57% male). A total of 549 KTR (82%) had a detectable TTV load (3.1 ± 1.5 log_10_ copies/mL). In KTR with a detectable TTV load, cyclosporin and tacrolimus use were positively associated with TTV load (St. β = 0.46, *p* < 0.001 and St. β = 0.66, *p* < 0.001, respectively), independently of adjustment for potential confounders. Current smoking and alcohol intake of >20 g/day were negatively associated with TTV load (St. β = −0.40, *p* = 0.004 and St. β = −0.33, *p* = 0.009, respectively), independently of each other and of adjustment for age, sex, kidney function, time since transplantation and calcineurin inhibitor use. This strong association of smoking and alcohol intake with TTV suggests a need to account for the smoking status and alcohol intake when applying TTV guided immunosuppression in KTR.

## 1. Introduction

In kidney transplant recipients (KTRs), immunosuppressive drugs are crucial to reduce the risk of organ rejection. A delicate balance in dosing of immunosuppressive medication is necessary to prevent either rejection of the graft in the case of inadequately low levels of immunosuppression, or infectious disease and cancer in the case of excessive immunosuppression. In clinical routine, monitoring the degree of immunosuppression post transplantation relies mainly on measuring trough levels of calcineurin inhibitors. However, these levels do not accurately reflect the risk of under- or over-immunosuppression, but rather correlate to drug-related toxicity [1]. Currently, no appropriate diagnostic test for optimal guidance in the dosing of the immunosuppressive regimen is available in routine clinical practice [2].

Monitoring Torque Teno Virus (TTV) in KTR is a promising strategy for quantifying immune function. TTV is a non-pathogenic, single-stranded DNA virus that is detectable in up to 94% of the general population [3]. The virus appears insensitive to conventional antiviral therapy used for CMV prophylaxis in the post-transplantation setting [4,5]. Higher activity of the immune system and risk of rejection are linked to a lower TTV load, whereas a decreased or suppressed immune function and risk of infection are linked to a higher TTV load [5,6,7,8,9,10,11,12]. Moreover, at later stages after transplantation, a high TTV load is associated with higher rates of all-cause mortality and higher rates of mortality due to infectious disease in kidney transplant recipients [13].

Altogether, quantifying immune function by monitoring TTV load could potentially be a novel tool for guiding personalized immunosuppressive therapy in transplant recipients. In order to use TTV guided immunosuppression in routine clinical practice, it is important to identify factors other than immunosuppressive medication that influence the TTV load. Since active smoking and alcohol intake are known to affect the immune system in various ways, which are not completely understood [14,15,16,17], we hypothesized that these behaviors could influence the TTV load. In this cross-sectional cohort study, we aimed to investigate the hypothesis that TTV load is associated with smoking and alcohol intake.

## 2. Materials and Methods

### 2.1. Study Population

For this cross-sectional study, we used data from the previously described prospective TransplantLines Food and Nutrition Biobank and Cohort study (ClinicalTrials.gov; #NCT02811835) [18]. In summary, KTRs (aged ≥ 18 years) at the University Medical Center Groningen (UMCG) with a functioning graft at least 1 year post transplantation, without active malignancies or infections and without a history of alcohol and/or drug abuse, were considered eligible for enrolment. Patients were included between November 2008 and June 2011 and provided written informed consent. The study was conducted according to the guidelines stated in the Declaration of Helsinki and the Declaration of Istanbul on Organ Trafficking and Transplant Tourism. The Institutional Review Board of the UMCG approved the study protocol (METc 2008/186). Participants were invited to the outpatient clinic, where all data were collected during a single visit. For our analyses, we excluded 40 subjects because of missing data on TTV load.

### 2.2. Assessment of Covariates

Medication use and medical history were extracted from patient records and were verified with the patients. Patients were weighed and measured wearing indoor clothing without shoes. Blood pressure and heart rate were measured with a semiautomatic device (Dinamap 1846; Critikon, Tampa, FL, USA). Patients were asked to collect 24 h urine the day before the visit. Blood was drawn in the morning after a fasting period of 8 to 12 h. Routine laboratory measurements were measured according to UMCG standard laboratory methods. Kidney function was assessed according to the CKD-EPI equation, based on creatinine and cystatin C [19]. Information regarding physical activity was inquired about using the short questionnaire to assess health-enhancing physical activity (SQUASH) [20,21].

### 2.3. TTV Load Measurements

Serum samples were stored at −80 °C until analyses. DNA was extracted from thawed serum using the eMAG Nucleic Acid Extraction System (bioMerieux, Marcy, France). The TTV R-GENE Real-time PCR assay was used on an Applied Biosystems 7500 (Thermo Fisher, Waltham, MA, USA) according to the manufacturer’s instructions, to measure the TTV load. A 1:4 dilution using DMEM was performed prior to sample extraction (ThermoFisher, Waltham, MA, USA). A cycle time above 40 was considered the upper limit of detection. The TTV R-GENE PCR assay is designed to detect TTV genotypes 1, 3, 6, 7, 8, 10, 12, 15, 16, 19, 27 and 28, covering the twelve genotypes that are a substantial component of the human virome [7,22,23]. TTV load is presented as log_10_ TTV copies per mL. TTV measurements and calculations were performed under ISO15189 guidelines [24].

### 2.4. Assessment of Smoking Status

Smoking status was self-reported using a questionnaire. Furthermore, cotinine was measured in 24 h urine samples. Samples were stored at −80 °C until assessment of cotinine. Cotinine concentrations were measured using the Immulite 2500 assay (Siemens, Los Angeles, CA, USA) with the intra- and interassay coefficient of variation ranging from 2.2% to 5.7%. To mitigate potential response bias and enhance the robustness of our analysis, the classification of active smoking was approached through various methods. This included questionnaire responses, cotinine measurements, and combinations thereof: self-reported smoking status; self-reported smoking status with addition of KTR with urinary cotinine < 0 ng/L (current smoker is self-reported and/or urinary cotinine < 0 ng/L); self-reported smoking status with addition of KTR with a urinary cotinine excretion of >50 ng/L (current smoker is self-reported and/or urinary cotinine < 50 ng/L); self-reported smoking status, excluding KTR with urinary cotinine <50 ng/L (current smoker is self-reported and urinary cotinine < 50 ng/L); self-reported smoking status, excluding KTR without urinary cotinine excretion (current smoker is self-reported and urinary cotinine < 0 ng/L); urinary cotinine excretion of > 0 ng/L (self-reported smoking status not taken into account); urinary cotinine excretion of >50 ng/L (self-reported smoking status not taken into account).

### 2.5. Assessment of Alcohol Intake

A validated food frequency questionnaire (FFQ) was used for the assessment of alcohol intake [25]. The FFQ contains questions on the intake of 177 different food and beverage items during the last month. All questionnaires were filled out at home by patients shortly prior to the study visit and checked for abnormalities by researchers. In the case of inconsistencies or abnormalities in the FFQ, patients were asked for a clarification. Dietary data were converted into energy and nutrient intake by research dieticians and nutritionists using the Dutch Food Composition Table [26]. For analyses, daily alcohol intake was categorized into above and below an intake of 2 alcoholic units (20 g) per day. Ethyl glucuronide (EtG), a direct metabolite of ethanol and hence a biomarker of alcohol consumption with a detection time up to 72 h after consumption, was measured in 24 h urine samples. The samples were stored at −80 °C until analyses. EtG concentrations were measured using the Thermo Scientific DRI Ethyl Glucuronide assay. The detection limit of this assay is 100 ng/mL and it has shown good agreement with established liquid chromatography/mass spectrometry methods in detecting EtG. Previously, the intra-assay coefficient of variation was established at <1.7% and the interassay coefficient of variation was established at <2.2% [27]. The concentration of ethyl glucuronide in urine was binary-categorized in both logistic and linear regression models, using a threshold value of 10,000 ug/L, which was selected as an arbitrary reference point.

### 2.6. Data Analysis

R statistical software was used for most analyses [28]. In all tables, variables with >3 missing values are reported in the footnotes. A two-sided *p* < 0.05 was considered statistically significant in all analyses.

### 2.7. Population Characteristics

Patients were stratified into two groups: KTR with no detectable TTV load and KTR with a detectable TTV load. Variables were tested for normality by visual inspection of histograms and Q-Q plots. Normally distributed variables were presented as the mean ± standard deviation, non-normally distributed variables were presented as the median [interquartile range], and nominal data as the number (valid percentage). For normally distributed variables, means between groups were compared using an independent *t*-test combined with Levene’s Test for Equality of Variances. For comparing non-normally distributed variables between groups, a Mann–Whitney U test was used. A chi-square test was used for comparing proportions of categorical variables.

### 2.8. Logistic Regression Analyses

Logistic regression analyses were performed, with a detectable or undetectable TTV load as a binary outcome serving as the dependent variable, and we performed additional sensitivity analyses with a TTV load < 4 log_10_ copies/mL or ≥4 log_10_ copies/mL as a binary outcome serving as the dependent variables. Univariable and multivariable logistic regression analyses were performed, the latter with cumulative adjustment for potential confounders including sex, age, time after transplantation, cystatin C-based eGFR, use of tacrolimus and use of cyclosporin. Cystatin C-based eGFR was chosen as it had the strongest association with TTV load among renal function measures. Odds ratios (ORs) for continuous variables were presented per standard deviation increase, to allow for comparison of effect sizes across independent variables.

### 2.9. Linear Regression Analyses

To assess the associations of smoking status and alcohol intake with TTV load, linear regression analyses were performed in the subgroup of KTR with a detectable TTV load, with TTV load serving as the dependent variable. Univariable and multivariable linear regression analyses were performed, the latter with cumulative adjustment for potential confounders including sex, age, time since transplantation (log_10_ transformed), cystatin C-based eGFR, use of tacrolimus and use of cyclosporin. Separate sensitivity analyses were performed to address potential outliers. All linear regression analyses described above were repeated while excluding KTR with the highest 2.5% TTV loads. Outcomes were presented as standardized β values. For continuous independent variables, regression coefficients were presented per standard deviation increase.

## 3. Results

### 3.1. Population Characteristics

**Figure 1:**
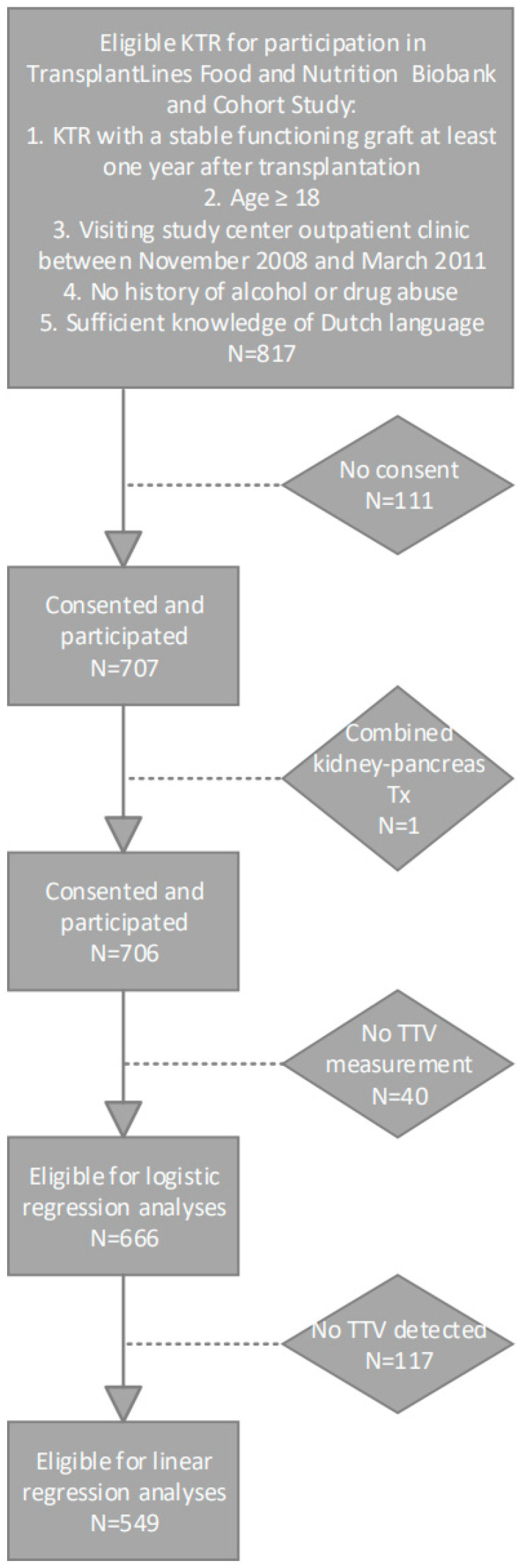
Flowchart of study population selection.

A total of 666 KTRs were included, of whom 549 (82%) had a detectable TTV load. The selection of the population is presented in Figure 1. Population characteristics for KTR with and KTR without a detectable TTV load are presented in Table 1. In KTR with a detectable TTV load, the mean TTV load was 3.05 ± 1.53 log_10_ copies/mL. Compared to KTRs without detectable TTV, KTRs with detectable TTV were older (54.0 ± 12.3 vs. 48.5 ± 14.3 years), had a shorter time since transplantation (5.1 [1.7–11.4] vs. 7.1 [4.1–12.4] years) and had a lower cystatin C-based eGFR (40.0 ± 18.2 vs. 46.4 ± 20.4 mL/min/1.73 m^2^). KTR with detectable TTV had a higher percentage of patients using CNI (*n* = 335 (61.0%) vs. *n* = 42 (35.9%)), mainly due to a higher percentage of patients using cyclosporin (*n* = 235 (42.8%) vs. *n* = 24 (20.5%)).

### 3.2. Logistic Regression Analyses

The results of the logistic regression analyses with a measurable TTV load (compared to no measurable TTV load) as the dependent variable are presented in Supplementary Table S1, and showed similar associations to the differences highlighted in the population characteristics.

The results of sensitivity analyses with a TTV load of 4 log_10_ copies/mL as the cut-off point are presented in Table S2. Smokers were less likely to have a TTV load ≥ 4 log_10_ copies/mL (OR: 0.43 [0.20–0.87]), independently of adjustment for age, sex, cystatin C-based eGFR, time since transplantation and use of calcineurin inhibitors. There was a trend for KTR with an alcohol intake above 20 g/day toward a decreased likelihood to have a TTV load ≥ 4 log_10_ copies/mL (OR: 0.50 [0.23–1.01]) although this association did not reach statistical significance. KTRs with an ethyl glucuronide concentration above 10.000 ug/L were less likely to have a TTV load ≥ 4 log_10_ copies/mL (OR: 0.39 [0.16–0.80]), independently of adjustment for age, sex, cystatin C-based eGFR, time since transplantation and use of calcineurin inhibitors. 

### 3.3. Linear Regression Analyses

The results of the linear regression analyses in KTR with detectable TTV are presented in Table 2.

**Figure 2:**
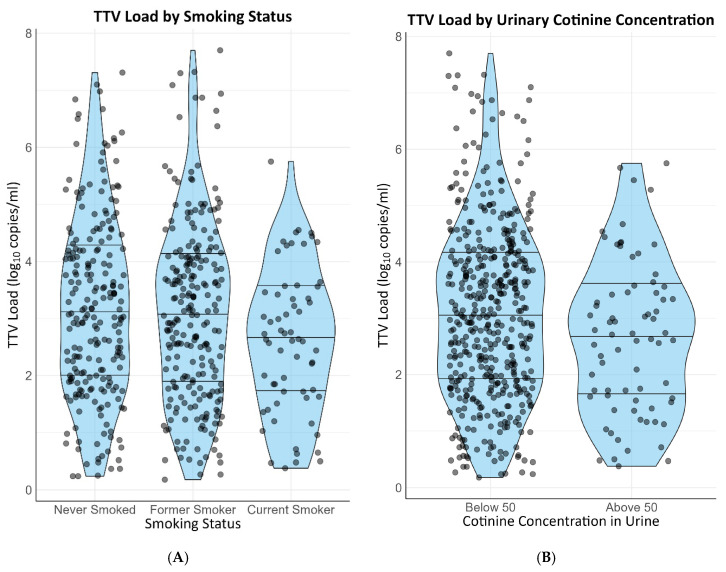
Distribution of TTV loads by smoking status. (**A**) Self-reported smoking status. (**B**) Measurable vs. unmeasurable cotinine in urine.

**Figure 3:**
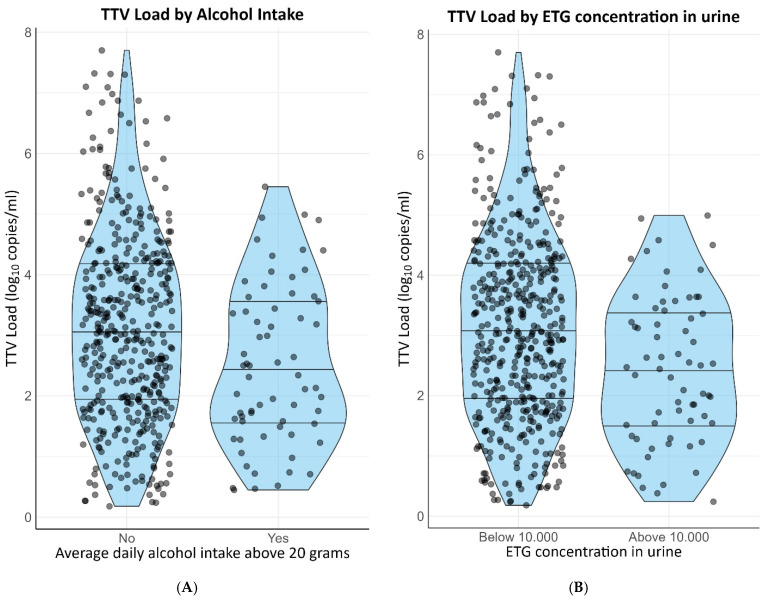
Distribution of TTV loads by alcohol intake. (**A**) Self-reported alcohol intake. (**B**) Urinary ETG concentration >10,000 ug/L.

Self-reported active smoking (β = −0.38 [−0.66–−0.10]) and self-reported alcohol intake of two or more units per day (st. β = −0.40 [−0.66–−0.14]) were negatively associated with TTV load. Both associations remained materially unchanged after adjusting for age, sex, cystatin C-based eGFR, time since transplantation and use of calcineurin inhibitors. Urinary cotinine and ethyl glucuronide, objective markers for smoking and alcohol intake, respectively, were also associated with TTV load. A measurable urinary cotinine concentration was negatively associated with TTV load (st. β = −0.28 [−0.51–−0.04]) and a concentration of ethyl glucuronide in the urine exceeding 10.000 ug/L also exhibited a negative association with TTV load (st. β = −0.45 [−0.71–−0.20]). Both current smoking and former smoking were associated with a higher alcohol intake on a continuous scale (st. β = 0.48 and st. β = 0.86, respectively). Nevertheless, the associations of current smoking and alcohol with TTV load were independent of each other. Distributions of TTV loads by smoking status are presented in Figure 2A,B. Distributions of TTV loads by alcohol intake are presented in Figure 3A,B.

Tacrolimus and cyclosporin use were positively associated with TTV load (st. β = 0.66 [0.43–0.89] and st. β = 0.46 [0.28–0.64], respectively). These associations remained when adjusting for age, sex, cystatin C-based eGFR and time since transplantation.

Time since transplantation (log_10_ transformed) was negatively associated with TTV (st. β = −0.24 [−0.32–−0.16]) and age was positively associated with TTV (st. β = 0.07 [0.02–0.15]), both independently of adjustment for age, sex, cystatin C-based eGFR and use of calcineurin inhibitors. Renal function as measured by cystatin C-based eGFR was negatively associated with TTV load (st. β = −0.21 [−0.29–−0.13]). The associations of cystatin C-based eGFR remained when adjusting for age, sex, time since transplantation and use of calcineurin inhibitors. Sex of the recipient was not associated with TTV load.

In sensitivity analyses when leaving out the highest 2.5% TTV loads, some of the associations lost significance. Results of these sensitivity analyses are presented in Supplementary Table S3.

## 4. Discussion

We hypothesized that smoking and alcohol intake are associated with TTV load. This study shows that smoking and alcohol consumption are strongly and independently associated with a lower TTV load, with effect sizes on TTV load that are similar to that of calcineurin inhibitor use. These findings suggest that potential effects of smoking and alcohol could be clinically relevant when using TTV load as a marker for immunosuppression in clinical practice.

Existing literature on smoking and alcohol’s relation to TTV load is limited. Spandole et al. found no association of smoking and alcohol intake with detectable versus undetectable TTV (among other anelloviruses) in the circulation of the general population of Romania [29]. Spandole et al. did not look into the viral load of TTV. In another study, Abbas et al. found no association between tobacco exposure and TTV load in bronchoalveolar fluid in pre-transplantation donor lungs [30], which appears to be inconsistent with our findings. However, it is unknown whether TTV load in bronchoalveolar fluid is a reflection of serum TTV load and it may be influenced differently by cigarette smoking. Furthermore, Abbas et al. investigated material from a vastly different population, and analyzing samples after procurement of lungs for transplantation might come with many other confounding factors. Our study is the first to find a negative association of smoking status and alcohol intake with serum TTV load. We propose several potential underlying mechanisms.

Smoking is known to exert complex and paradoxical effects on immune cells, for instance, altering the expression and functionality of different T-cells [17]. Since TTV is influenced by T-cells, it is conceivable that smoking-induced immunological changes could alter TTV load. Is has been suggested that TTV replicates mainly in T-cells, and it has previously been shown that a temporary decrease in the number of T-cells due to induction therapy shortly post-transplant is accompanied by a concurrent drop in TTV load [31]. Regulation of TTV replication in T-cells is not yet clarified, but effects of smoking on T-cells may decrease the replication of TTV in T-cells. Alternatively, it is believed that the host response against TTV is also mainly T-cell-driven [32], and previous research has shown that TTV replication is inversely correlated with the number and functionality of T-cells [33,34]. It seems unlikely that smoking would improve immune function; however, it is theoretically possible that TTV load may be decreased due to a better control of the virus by the immune system. This possibility could be investigated in future research by measuring other markers for T-cell function, such as the Quantiferon-Monitor [35].

Conflicting effects of alcohol intake on the immune system have been reported. Both cell-mediated and humoral immune responses, as well as the expression of different cytokines, are altered by alcohol consumption [14,16]. The exact mechanisms behind the opposing effects of alcohol on the immune system are complex and incompletely understood [14,16]. There is evidence to suggest that some compounds in polyphenolic-rich alcoholic beverages, including wine and beer, could have an immune stimulating effect in the context of low-to-moderate alcohol consumption, which could lead to better control of the virus [15].

Furthermore, alcohol or chemicals from tobacco smoke may directly harm TTV or compete with TTV. Several compounds of cigarette smoke have been shown to have antiviral properties, such as arsenic [36], carbon monoxide [37], formaldehyde [38] and cadmium [39]. It should, however, also be realized that cigarette smoking has been associated with higher viral loads in patients with human immunodeficiency virus [40], which suggests that an effect of cigarette smoke exposure on concentrations of viruses, if present, is not the same for every virus.

Moreover, smoking influences pharmacokinetics and/or pharmacodynamics of several drugs, for instance, due to effects of tobacco smoke on several CYP enzymes. Calcineurin inhibitors are primarily metabolized by CYP3A4 and CYP3A5 [41,42]. To the best of our knowledge, not much is known about the effect of tobacco smoking on the activity of CYP3A4 and CYP3A5 in humans. In a rat model, there was a potential inhibitory effect on CYP3A4 [43], which could lead to higher calcineurin inhibitor levels and would contradict our findings.

Tobacco smoke is known to cause adducts on DNA, which precede DNA mutations if not repaired before DNA replication. Chronic alterations in the DNA sequence can occur when adducted DNA is replicated [44,45,46]. It is theoretically possible that DNA adducts cause altered replication of the virus during the PCR process, leading to distorted results of the assay. To the best of our knowledge, the effect of DNA adducts on PCR results has not been studied.

Additionally, KTRs who smoke and drink might represent a healthier subset of the population with accompanying better overall immune function. These behaviors may be tolerated better physically in healthy KTR than in individuals with poor health conditions, and might in addition be a measure of social behavior, which is likely to be more present in healthier KTR.

An important strength of this study is our large and well-characterized study population of KTR. Furthermore, TTV was measured using a standardized assay, the TTV R-GENE kit [21], in order to obtain reproducible TTV measurements. For the assessment of smoking behavior and alcohol intake, both self-reported and objective measures were used for internal validation. In addition, our findings were robust after adjustment for multiple potential confounders and in sensitivity analyses. Because of the selection of our population, the results of our study were unaffected by the dynamic changes in TTV load in the first months following transplantation, since previous studies have shown that TTV load rapidly increases following organ transplantation, before stabilizing or slowly declining after 3 months [7,47].

Some limitations of the study need to be addressed. In total, 82% of our population had a detectable TTV load, which is in line with existing literature [3,6,48]. However, an undetectable TTV load can either represent an uninfected individual (true negative) or can be the consequence of a strong immune function suppressing the TTV load below the limit of detection, resulting in a negative PCR result. Ideally, one would only exclude the true negatives from all linear regression analyses. Unfortunately, within our data, there is no definitive way to determine which negative TTV measurements are truly negative due to absence of prior exposure, and which measurements are negative due to strong suppression of TTV. Therefore, all TTV negative KTRs, including a number of TTV infected KTRs, were left out of our linear regression analyses.

Future studies are needed to investigate the causality and the etiology of the association of current smoking and alcohol intake with TTV load. In the current study, it remains unclear whether these associations represent clinically relevant shifts in immune function that influence the chances of graft rejection or infection, or reflect an effect of smoke exposure and alcohol intake on the TTV load without substantially altering immune function, which should be corrected for when using TTV guided immunosuppression. Furthermore, it would be valuable to validate our findings in KTR within the first year after transplantation, in which dynamic changes in TTV loads occur and in which TTV guided immunosuppression is currently being investigated in randomized controlled trials [6,33,49].

In conclusion, smoking and alcohol intake are associated with a lower circulating TTV load in stable kidney transplant recipients with an effect size similar to that of calcineurin inhibitors. Our study suggests a potential need to account for smoking status and alcohol intake when applying TTV guided immunosuppression in KTRs.

## Figures and Tables

**Table 1:** Population characteristics in patients with and without detectable TTV load.

Variable	Undetectable TTV (n = 117)	Detectable TTV (n = 549)	*p*-Value
TTV load (log_10_ copies/mL), mean (SD)	NA	3.05 (1.53)	NA
Clinical characteristics			
Female sex, n (%)	60 (51.3)	228 (41.5)	0.05
Age (years), mean (SD)	48.5 (14.3)	54.0 (12.26)	<0.001
Height (cm), mean (SD)	172 (10)	174 (10)	0.13
Weight (kg), mean (SD)	77.3 (15.1)	81.1 (16.5)	0.02
BMI (kg/m^2^), mean (SD)	26.0 (4.3)	26.8 (4.8)	0.09
Systolic blood pressure (mmHg), mean (SD)	134 (18)	137 (17.4)	0.10
History of diabetes, n (%)	22 (18.8)	138 (25.1)	0.15
Transplantation characteristics			
Living donor, n (%)	45 (38.5)	187 (34.1)	0.36
Age of the donor (years), mean (SD)	40.6 (15.3)	43.5 (15.4)	0.08
Female sex of the donor, n (%)	58 (53.7)	257 (47.3)	0.23
Time since transplantation (years), median [IQR]	7.1 [4.1, 12.4]	5.1 [1.7, 11.4]	0.009
Positive CMV serostatus of the donor, n (%)	43 (42.6)	243 (47.3)	0.46
Positive CMV serostatus of the recipient, n (%)	24 (30.8)	147 (40.9)	0.22
Laboratory measurements			
Hemoglobin (mmol/L), mean (SD)	8.2 (1.1)	8.3 (1.1)	0.80
Leukocyte count (10^9^/L), mean (SD)	8.0 (2.4)	8.2 (2.6)	0.43
Trombocyte count (10^9^/L), mean (SD)	241.8 (72.4)	234.9 (74.5)	0.57
Creatine (umol/L), median [IQR]	115 [91, 144]	126 [102, 163]	0.01
eGFR creatine (mL/min/1.73 m^2^), mean (SD)	57.4 (22.3)	51.3 (19.9)	0.003
Cystatin C (mg/L), median [IQR]	1.46 [1.22, 2.00]	1.71 [1.35, 2.28]	0.004
eGFR cystatin (mL/min/1.73 m^2^), mean (SD)	46.4 (20.4)	30.0 (18.2)	0.001
HS-CRP (mg/dL), median [IQR]	1.40 [0.65, 3.60]	1.60 [0.70, 4.60]	0.40
Albumin (g/L), mean (SD)	43.3 (2.9)	42.9 (3.0)	0.21
Total urinary protein excretion (g/24 h), median [IQR]	0.2 [0.0, 0.3]	0.2 [0.0, 0.4]	0.13
Lifestyle factors			
SQUASH score, median [IQR]	5700 [2780, 9060]	4935 [1920, 7500]	0.23
Average alcohol intake (grams/day), median [IQR]	4.1 [0.3, 11.8]	2.3 [0.0, 10.6]	0.22
Average daily alcohol intake above 20 g, n (%)	13 (12.5)	66 (13.2)	0.85
Ethyl glucuronide concentration in urine above 10.000 ug/L, n (%)	18 (15.5)	66 (12.5)	0.38
Smoking behavior according to questionnaire			0.42
- Never smoked, n (%)	42 (38.9)	221 (42.6)	
- Former smoker, n (%)	48 (44.4)	235 (45.3)	
- Current smoker, n (%)	18 (16.7)	63 (12.1)	
Smoking behavior correct for urinary cotinine above 0 ng/L			0.50
- Never smoked and no urinary cotinine, n (%)	41 (37.6)	203 (39.7)	
- Former smoker and no urinary cotinine, n (%)	41 (37.6)	207 (40.5)	
- Current smoker or measurable urinary cotinine, n (%)	27 (24.8)	101 (19.8)	
Cotinine concentration in urine above 0 ng/L, n (%)	26 (22.4)	86 (16.3)	0.11
Medication			
Calcineurin inhibitor usage			<0.001
- None, n (%)	75 (64.1)	214 (39.0)	
- Cyclosporin, n (%)	24 (20.5)	235 (42.8)	
- Tacrolimus, n (%)	18 (15.4)	100 (18.2)	
Prednisolone usage, n (%)	115 (98.3)	545 (99.3)	0.31
Proliferation inhibitor usage, n (%)	104 (88.9)	451 (82.1)	0.08
mTOR inhibitor usage, n (%)	3 (2.6)	19 (3.5)	0.62

Abbreviations: eGFR = estimated glomerular filtration rate as calculated using the creatinine and cystatin C-based CKD-EPI formula, hs-CRP = high-sensitivity C-reactive protein. Normally distributed data are presented as mean ± standard deviation, non-normally divided data as median [interquartile range], and categorical data as number (valid percentage). *p*-Values were calculated with independent *t*-test for normally distributed data, with Mann–Whitney U test for non-normally divided data, and with Chi-square test for categorical data. Missing data: age of the donor was missing for 18 patients, sex of the donor was missing for 15 patients, trombocyte count was missing for 409 patients, CRP was missing for 35 patients, alcohol intake was missing for 61 patients, CMV status of the recipient was missing for 229 patients, CMV status of the donor was missing for 51 patients, smoking behavior was missing for 39 patients, ethyl glucuronide in the urine was missing for 21 patients. All other variables had missing data for 0–3 patients.

**Table 2:** Results of univariable and multivariable linear regression analyses in KTR with a detectable TTV load (*n* = 549), with TTV load as dependent variable.

Variable	Univariable		Multivariable	
	St. β [95% CI]	*p*-Value	St. β [95% CI]	*p*-Value
Female sex	0.00 [−0.17–0.17]	0.99	−0.04 [−0.20–0.12]	0.64
Age	0.07 [−0.02–0.15]	0.12	0.10 [0.02–0.18]	0.02
Time since transplantation [log_10_]	−0.24 [−0.32–−0.16]	<0.001	−0.18 [−0.27–−0.10]	<0.001
eGFR cystatin	−0.21 [−0.29–−0.13]	<0.001	−0.15 [−0.23–−0.06]	0.001
Calcineurin inhibitor usage				
- None	Reference		Reference	
- Cyclosporin	0.46 [0.28–0.64]	<0.001	0.26 [0.07–0.45]	0.008
- Tacrolimus	0.66 [0.43–0.89]	<0.001	0.46 [0.21–0.71]	<0.001
Smoking variables				
Smoking questionnaire				
- Never smoked	Reference		Reference	
- Former smoker	−0.06 [−0.24–0.13]	0.55	−0.10 [−0.28–0.08]	0.26
- Current smoker	−0.38 [−0.66–−0.10]	0.008	−0.40 [−0.66–−0.13]	0.004
Smoking behavior corrected for urinary cotinine above 50 ng/L				
- Never smoked and low urinary cotinine	Reference		Reference	
- Former smoker and low urinary cotinine	−0.02 [−0.21–0.18]	0.87	−0.07 [−0.26–0.12]	0.48
- Current smoker or high urinary cotinine	−0.25 [−0.50–0.00]	0.048	−0.30 [−0.54–−0.06]	0.01
Smoking behavior corrected for urinary cotinine above 0 ng/L				
- Never smoked and no urinary cotinine	Reference		Reference	
- Former smoker and no urinary cotinine	−0.01 [−0.08–0.19]	0.91	−0.06 [−0.25–0.13]	0.52
- Current smoker or measurable urinary cotinine	−0.25 [−0.49–−0.01]	0.04	−0.29 [−0.52–−0.06]	0.01
Active smoking and urinary cotinine above 50 ng/L	−0.39 [−0.68–−0.10]	0.008	−0.39 [−0.67–−0.12]	0.005
Active smoking and urinary cotinine above 0 ng/L	−0.40 [−0.68–−0.12]	0.005	−0.39 [−0.65–−0.13]	0.004
Cotinine concentration in urine above 50 ng/L	−0.29 [−0.54–−0.04]	0.02	−0.32 [−0.55–−0.08]	0.009
Cotinine concentration in urine above 0 ng/L	−0.28 [−0.51–−0.04]	0.02	−0.30 [−0.52–−0.08]	0.008
Alcohol variables				
Average daily alcohol intake above 20 g/day	−0.40 [−0.66–−0.14]	0.003	−0.33 [−0.58–−0.08]	0.009
Ethyl glucuronide concentration in urine above 10.000 ug/L	−0.45 [−0.71–−0.20]	0.001	−0.35 [−0.60–−0.11]	0.005

Abbreviations: eGFR = estimated glomerular filtration rate as calculated using the creatinine and cystatin C-based CKD-EPI formula. Multivariable: adjusted for age, sex, eGFR with cystatin C-based CKD-EPI formula, time since transplantation (log_10_ transformed) and calcineurin inhibitor use. Missing data: smoking behavior was missing for 39 patients; alcohol intake was missing for 61 patients; ethyl glucuronide in the urine was missing for 21 patients. All other variables had missing data for 0–3 patients.

## Data Availability

The data underlying the results presented in this study can be made available by the data manager of the TransplantLines study, by mailing to transplantlines@umcg.nl or by contacting the corresponding author.

## Supplementary Materials

The following supporting information can be downloaded at: https://www.mdpi.com/article/10.3390/v15122387/s1, Table S1. Univariable and multivariable logistic regression analyses’ results, showing the odds ratios for having a detectable TTV load. Table S2. Univariable and multivariable logistic regression analyses’ results, showing the odds ratios for having a TTV load ≥ 4 log_10_ copies/mL. Table S3. Results of univariable and multivariable linear regression analyses in KTRs with a detectable TTV load wherein the patients with the top 2.5% TTV load are excluded (*n* = 535), with TTV load as dependent variable.
