# Supplementary material for: Smoking, Alcohol Intake and Torque Teno Virus in Stable Kidney Transplant Recipients
Source: Viruses. 2023 Dec 6;15(12):2387. doi: 10.3390/v15122387 (PMC10748022; doi:10.3390/v15122387)
Supplement: Supplementary file 1 [file viruses-15-02387-s001.zip › viruses-2756903-supplementary.pdf]

Supplementary Materials:

Table S1 Univariable and multivariable logistic regression analyses results, showing the odds ratios for having a detectable TTV load.

Variable	Univariable OR [95% CI]	*p*-Value	Multivariable OR [95% CI]	*p*-Value
Female sex	0.67 [0.45 – 1.01]	0.050	0.66 [0.43 – 1.00]	0.050
Age	1.51 [1.24 – 1.84]	<0.001	1.54 [1.26 – 1.91]	<0.001
Time since transplantation [$\log_{10}$]	0.74 [0.60 – 0.91]	0.004	0.82 [0.64 – 1.04]	0.11
eGFR cystatin	0.72 [0.59 – 0.87]	0.001	0.83 [0.68 – 1.03]	0.09
Calcineurin inhibitor usage				
- None	Reference		Reference	
- Cyclosporin usage	3.43 [2.12 – 5.73]	<0.001	2.83 [1.67 – 4.94]	<0.001
- Tacrolimus usage	1.95 [1.13 – 3.52]	0.02	1.70 [0.91 – 3.28]	0.11
Smoking variables				
Smoking questionnaire				
- Never smoked	Reference		Reference	
- Former smoker	0.93 [0.59 – 1.46]	0.76	0.68 [0.41 – 1.13]	0.14
- Current smoker	0.67 [0.36 – 1.26]	0.20	0.52 [0.27 – 1.00]	0.050
Smoking behaviour correct for urinary cotinine above 50 ng/L				
- Never smoked and low urinary cotinine	Reference		Reference	
- Former smoker and low urinary cotinine	1.00 [0.63 – 1.61	0.98	0.72 [0.43 – 1.22]	0.22
- Current smoker or high urinary cotinine	0.72 [0.42 – 1.27]	0.25	0.56 [0.31 – 1.02]	0.06
Smoking behaviour correct for urinary cotinine above 0 ng/L				
- Never smoked and no urinary cotinine	Reference		Reference	
- Former smoker and no urinary cotinine	1.02 [0.63 – 1.64]	0.94	0.72 [0.42 – 1.22]	0.23
- Current smoker or measurable urinary cotinine	0.76 [0.44 – 1.31]	0.31	0.60 [0.34 – 1.09]	0.09
Cotinine concentration in urine above 50 ng/L	0.68 [0.41 – 1.18]	0.16	0.66 [0.38 – 1.17]	0.15
Cotinine concentration in urine above 0 ng/L	0.67 [0.41 – 1.12]	0.12	0.64 [0.38 – 1.11]	0.10
Alcohol variables				
Average daily alcohol intake above 20 grams/day	1.06 [0.58 – 2.09]	0.85	1.02 [0.53 – 2.10]	0.95
Ethyl glucuronide concentration in urine above 10.000 ug/L	0.78 [0.45 – 1.40]	0.38	0.83 [0.46 – 1.53]	0.53

Abbreviations: eGFR = estimated glomerular filtration rate as calculated using the creatinine and cystatin C-based CKD-EPI formula. Multivariable: adjusted for age, sex, eGFR by cystatin C-based CKD-EPI formula, time since transplantation ($\log_{10}$ transformed) and calcineurin inhibitor use. Missing data: smoking behaviour was missing in 39 patients; alcohol intake was missing in 61 patients, ethyl glucuronide in the urine was missing for 21 patients. All other variables had missing data for 0-3 patients.

Table S2 Univariable and multivariable logistic regression analyses results, showing the odds-ratios for having a TTV load ≥4 log_{10} copies/mL.

**Variable**	**Univariable OR [95% CI]**	***p*-Value**	**Multivariable OR [95% CI]**	***p*-Value**
Female sex	0.89 [0.61 – 1.28]	0.52	0.85 [0.57 – 1.26]	0.42
Age	1.30 [1.08 – 1.58]	0.007	1.40 [1.13 – 1.74]	0.002
Time since transplantation [log_{10}]	0.52 [0.43 – 0.63]	<0.001	0.57 [0.46 – 0.71]	<0.001
eGFR cystatin	0.63 [0.51 – 0.77]	<0.001	0.70 [0.55 – 0.87]	0.002
Calcineurin inhibitor usage				
- None	Reference		Reference	
- Cyclosporin usage	2.85 [1.84 – 4.49]	<0.001	1.61 [0.97 – 2.68]	0.06
- Tacrolimus usage	3.86 [2.31 – 6.52]	<0.001	2.38 [1.31 – 4.30]	0.004
Smoking variables				
Smoking questionnaire				
- Never smoked	Reference		Reference	
- Former smoker	0.85 [0.58 – 1.27]	0.43	0.67 [0.43 – 1.05]	0.08
- Current smoker	0.46 [0.22 – 0.89]	0.03	0.43 [0.20 – 0.87]	0.02
Smoking behaviour correct for urinary cotinine above 50 ng/L				
- Never smoked and low urinary cotinine	Reference		Reference	
- Former smoker and low urinary cotinine	0.89 [0.59 – 1.34]	0.58	0.68 [0.42 – 1.07]	0.10
- Current smoker or high urinary cotinine	0.60 [0.33 – 1.05]	0.08	0.54 [0.29 – 0.98]	0.049
Smoking behaviour correct for urinary cotinine above 0 ng/L				
- Never smoked and no urinary cotinine	Reference		Reference	
- Former smoker and no urinary cotinine	0.92 [0.60 – 1.39]	0.68	0.69 [0.43 – 1.11]	0.13
- Current smoker or measurable urinary cotinine	0.60 [0.34 – 1.03]	0.07	0.55 [0.30 – 0.98]	0.048
Active smoking and urinary cotinine above 50 ng/L	0.43 [0.19 – 0.88]	0.03	0.44 [0.19 – 0.93]	0.045
Active smoking and urinary cotinine above 0 ng/L	0.45 [0.20 – 0.88]	0.03	0.46 [0.20 – 0.94]	0.04
Cotinine concentration in urine above 50 ng/L	0.56 [0.30 – 1.00]	0.06	0.58 [0.29 – 1.06]	0.09
Cotinine concentration in urine above 0 ng/L	0.58[0.32 – 0.98]	0.05	0.60 [0.32 – 1.06]	0.09
Alcohol variables				
Average daily alcohol intake above 20 grams/day	0.46 [0.22 – 0.89]	0.03	0.50 [0.23 – 1.01]	0.07
Ethyl glucuronide concentration in urine above 10.000 ug/L	0.33 [0.14 – 0.36]	0.004	0.39 [0.16 – 0.80]	0.02

Abbreviations: eGFR = estimated glomerular filtration rate as calculated using the creatinine and cystatin C-based CKD-EPI formula. Multivariable: adjusted for age, sex, eGFR by cystatin C-based CKD-EPI formula, time since transplantation (log_{10} transformed) and calcineurin inhibitor use. Missing data: smoking behaviour was missing in 39 patients; alcohol intake was missing in 61 patients, ethyl glucuronide in the urine was missing for 21 patients. All other variables had missing data for 0-3 patients.

Table S3 Results of univariable and multivariable linear regression analyses in KTR with a detectable TTV load wherein the patients with the top 2.5% TTV load are excluded (n=535), with TTV load as dependent variable.

**Variable**	**Univariable**		**Multivariable**	
	**St. β [95% CI]**	***p*-Value**	**St. β [95% CI]**	***p*-Value**
Female sex	-0.07 [-0.25 -0.10]	0.40	-0.11 [-0.28 – 0.05]	0.18
Age	0.09 [0.01 – 0.18]	0.03	0.11 [0.02 – 0.19]	0.01
Time since transplantation [log_{10}]	-0.21 [-0.29 – -0.12]	<0.001	-0.16 [-0.25 – -0.07]	<0.001
eGFR cystatin	-0.20 [-0.29 – -0.12]	<0.001	-0.16 [-0.24 – -0.07]	<0.001
Calcineurin inhibitor usage				
- None	Reference		Reference	
- Cyclosporin	0.46 [0.27 – 0.64]	<0.001	0.28 [0.08 – 0.47]	0.006
- Tacrolimus	0.44 [0.20 – 0.68]	<0.001	0.26 [0.00 – 0.52]	0.051
Smoking variables				
Smoking questionnaire				
- Never smoked	Reference		Reference	
- Former smoker	-0.05 [-0.27 – 0.10]	0.36	-0.14 [-0.33 – 0.04]	0.13
- Current smoker	-0.34 [-0.62 – -0.06]	0.02	-0.37 [-0.64 – -0.10]	0.008
Smoking behaviour correct for urinary cotinine above 50 ng/L				
- Never smoked and low urinary cotinine	Reference		Reference	
- Former smoker and low urinary cotinine	-0.05 [-0.25 – 0.14]	0.61	-0.11 [-0.30 – 0.08]	0.26
- Current smoker or high urinary cotinine	-0.19 [-0.44 – 0.05]	0.13	-0.26 [-0.50 – -0.02]	0.04
Smoking behaviour correct for urinary cotinine above 0 ng/L				
- Never smoked and no urinary cotinine	Reference		Reference	
- Former smoker and no urinary cotinine	-0.04 [-0.24 – 0.15]	0.66	-0.11 [-0.30 – 0.09]	0.29
- Current smoker or measurable urinary cotinine	-0.19 [-0.43 – 0.05]	0.13	-0.25 [-0.49 – -0.02]	0.03
Cotinine concentration in urine above 50 ng/L	-0.22 [-0.47 – 0.02]	0.08	-0.27 [-0.51 – -0.03]	0.03
Cotinine concentration in urine above 0 ng/L	-0.21 [-0.44 – 0.02]	0.08	-0.25 [-0.47 – -0.03]	0.03
Alcohol variables				
Average daily alcohol intake above 20 grams/day	-0.35 [-0.61 – -0.09]	0.009	-0.33 [-0.58 – -0.08]	0.01
Ethyl glucuronide concentration in urine above 10.000 ug/L	-0.41 [-0.66 – -0.15]	0.002	-0.34 [-0.58 – -0.09]	0.008

Abbreviations: eGFR = estimated glomerular filtration rate as calculated using the creatinine and cystatin C-based CKD-EPI formula. Multivariable: adjusted for age, sex, eGFR by cystatin C-based CKD-EPI formula, time since transplantation (log_{10} transformed) and calcineurin inhibitor use. Missing data: smoking behaviour was missing in 30 patients; alcohol intake was missing in 47 patients, ethyl glucuronide in the urine was missing for 20 patients. All other variables had missing data for 0-3 patients.
